# Case report: Long-lasting SARS-CoV-2 infection with post-COVID-19 condition in two patients with chronic lymphocytic leukemia: The emerging therapeutic role of casirivimab/imdevimab

**DOI:** 10.3389/fonc.2022.945060

**Published:** 2022-09-30

**Authors:** Laura Ballotta, Omar Simonetti, Pierlanfranco D’Agaro, Ludovica Segat, Raffaella Koncan, Pamela Martinez-Orellana, Federica Dattola, Emanuele Orsini, Alessandro Marcello, Simeone Dal Monego, Danilo Licastro, Andrea Misin, Sara Mohamed, Eugenio Sbisà, Elisa Lucchini, Giovanni Maria De Sabbata, Francesco Zaja, Roberto Luzzati

**Affiliations:** ^1^ Dipartimento Clinico di Scienze Mediche, Chirurgiche e della Salute, Università degli Studi di Trieste, Trieste, Italy; ^2^ Unità Complessa Operativa (UCO) Ematologia, Azienda Sanitaria Universitaria Giuliano Isontina, Trieste, Italy; ^3^ Struttura Complessa (SC) Malattie Infettive, Azienda Sanitaria Universitaria Giuliano Isontina, Trieste, Italy; ^4^ Unità Complessa Operativa (UCO) Igiene e Sanità Pubblica, Azienda Sanitaria Universitaria Integrata Giuliano Isontina, Trieste, Italy; ^5^ Laboratory of Molecular Virology, International Centre for Genetic Engineering and Biotechnology (ICGEB), AREA Science Park, Trieste, Italy; ^6^ Department of Life Sciences, Università degli Studi di Trieste, Trieste, Italy; ^7^ Biomedicine, AREA Science Park, Trieste, Italy

**Keywords:** chronic lymphocyte leukemia, COVID - 19, monoclonal antibodies, casirivimab/imdevimab, post COVID-19 condition

## Abstract

Post-coronavirus disease 2019 (post-COVID-19) condition, previously referred to as long COVID, includes a post-acute syndrome defined by the presence of non-specific symptoms occurring usually 3 months from the onset of the acute phase and lasting at least 2 months. Patients with chronic lymphocytic leukemia (CLL) represent a high-risk population for COVID-19. Moreover, the response to SARS-CoV-2 vaccination is often absent or inadequate. The introduction of monoclonal antibodies (mAbs) in the treatment landscape of COVID-19 allowed to reduce hospitalization and mortality in mild–moderate SARS-CoV-2 infection, but limited data are available in hematological patients. We here report the effective use of casirivimab/imdevimab (CI) in the treatment of two CLL patients with persistent infection and post-COVID-19 condition. Full genome sequencing of viral RNA from nasopharyngeal swabs was performed at the time of COVID-19 diagnosis and before the administration of CI. Both patients experienced persistent SARS-CoV-2 infection with no seroconversion for 8 and 7 months, respectively, associated with COVID symptoms. In both cases after the infusion of CI, we observed a rapid negativization of the nasal swabs, the resolution of post-COVID-19 condition, and the development of both the IgG against the trimeric spike protein and the receptor-binding domain (RBD) of the spike protein. The analysis of the viral genome in the period elapsed from the time of COVID-19 diagnosis and the administration of mAbs showed the development of new mutations, especially in the *S* gene. The genome variations observed during the time suggest a role of persistent SARS-CoV-2 infection as a possible source for the development of viral variants. The effects observed in these two patients appeared strongly related to passive immunity conferred by CI treatment permitting SARS-CoV-2 clearance and resolution of post-COVID-19 condition. On these grounds, passive anti-SARS-CoV-2 antibody treatment may represent as a possible therapeutic option in some patients with persistent SARS-CoV-2 infection.

## Introduction

Although most of the patients affected by the coronavirus disease 2019 (COVID-19) fully recovered within a few weeks, a large proportion of them, up to 76% at 6 months, reported at least one symptom of a post-COVID-19 condition (1). Post-COVID-19 condition (previously referred to as long COVID) occurs in individuals with a history of probable or confirmed severe acute respiratory syndrome coronavirus 2 (SARS-CoV-2) infection usually 3 months from the onset of COVID-19, with symptoms that last for at least 2 months and cannot be explained by an alternative diagnosis. The common symptoms include fatigue, shortness of breath, and cognitive dysfunction among others and generally have an impact on everyday functioning. The symptoms may be new onset following initial recovery from an acute COVID-19 episode or persist from the initial illness, and these symptoms may also fluctuate or relapse over time (2).

Patients with hematological malignancies represent a high-risk population for COVID-19 because of immunodeficiency related to their disease (e.g., hypogammaglobulinemia, dysfunction of the innate and adaptive immune system), immunosuppressive therapies (3), and frequent hospital admissions. A recent meta-analysis on 3,337 hematologic patients with COVID-19 demonstrated a death risk of 34% for adult patients and 4% for pediatric patients. Additionally, the pooled risk of death for lymphoma and chronic lymphocytic leukemia (CLL) patients was 32% and 31%, respectively (4). Furthermore, adult patients with cancer, including hematological patients, are likely to develop post-COVID-19 complications, particularly respiratory symptoms and residual fatigue, in about 15% of the cases with high rates of cessation or modification of anticancer treatment and subsequent impairment of prognosis and survival (5).

Based on these data, hematological patients represent a high-priority population for vaccination in order to mitigate COVID-19 morbidity and mortality. Unfortunately, the experiences of these patients resulted in suboptimal antibody responses following COVID-19 vaccination. For instance, the serologic response to the BNT162b2 mRNA COVID-19 vaccine was about 40% in CLL patients with a lower response rate in patients actively treated with Bruton’s tyrosine kinase inhibitors (BTKs) or venetoclax +/− anti-CD20 antibodies (6).

Since the beginning of the COVID-19 pandemic in 2020, the therapy for COVID-19 has focused on the acute viral phase of SARS-CoV-2 infection. The availability of an effective antiviral is even more crucial for unvaccinated patients as well as for immunocompromised patients not responding effectively to vaccination. Various therapeutic options have been explored for patients with COVID-19, including convalescent plasma and immunomodulators, with contrasting results (7–10). In the last year, the therapeutic strategies for COVID-19 have been enriched. With the aim to reduce viral load, prevent hospitalization, and ameliorate the symptoms of COVID-19, the U.S. Food and Drug Administration (FDA) and the European Medicines Agency (EMA) had issued the emergency use authorization (EUA) for monoclonal antibodies (mAbs) targeting the SARS-CoV-2 spike protein. In particular, the combination of bamlanivimab and etesevimab, casirivimab and imdevimab (REGN-COV2), and sotrovimab has been shown to prevent hospitalization and mortality in high-risk outpatients with mild-to-moderate COVID-19 (11–13). Though data regarding the use of mAbs in early COVID-19 cases are more robust (11–13), limited data on their use in immunocompromised patients with prolonged SARS-CoV-2 infection are available (14, 15). Here, we present our experience on the use of mAbs in the treatment of two patients affected by CLL and post-COVID-19 condition with long-term persistently positive SARS-CoV-2 infection.

## Case 1

On 17 January 2021, a 66-year-old man was admitted to the emergency department due to fever, asthenia, ageusia, anosmia, diarrhea, and dyspnea. This patient had a history of type 2 diabetes on metformin therapy and was followed by our hematological department since June 2019 for an untreated CLL associated with severe hypogammaglobulinemia. At diagnosis, the biologic characterization of CLL cells showed unmutated heavy chain variable (IGHV), and the fluorescence *in situ* hybridization (FISH) analysis was positive for trisomy of chromosome 12 with no further alterations of chromosomes 11, 13, and 17 and TP53 wild type. A nasal swab was positive for molecular testing [reverse transcription-polymerase chain reaction (RT-PCR)] of SARS-CoV-2. Arterial blood gas showed hypoxia with *p*O_2_ equal to 57 mmHg in ambient air, and chest X-ray and pulmonary computed tomography (CT) revealed a bilateral interstitial pneumonia. The patient was admitted to the Infectious Diseases (ID) unit for oxygen supply (e.g., high-flow nasal oxygen, HFNC), dexamethasone, and enoxaparin prophylaxis. At admission, his blood tests were as follows: white blood cells 44 × 10^9^/L, neutrophils 2 × 10^9^/L, lymphocytes 41 × 10^9^/L, hemoglobin 87 g/L, platelet count 256 × 10^9^/L, C-reactive protein 106.8 mg/L, D-dimer 2,597 ng/ml, total proteins 60 g/L with severe hypogammaglobulinemia (gamma globulin 6.9%, IgG 4.45 g/L), and stable mild paraproteinemia IgG lambda (1 g/L). During the subsequent period of hospitalization, the patient required a course of antibiotic therapy with piperacillin/tazobactam and gentamycin for hospital-acquired pneumonia. He improved progressively, and 30 days after the admission, the patient was discharged with nasal swabs RT-PCR-positive for SARS-CoV-2. During the following 5 months, the patient continued to complain of dysgeusia, anosmia, dyspnea, asthenia, and foot paraesthesia with a slight impairment in maintaining an erect posture and in walking. Such symptoms were attributable to the post-COVID-19 condition and required a multidisciplinary approach. Nasopharyngeal swab RT-PCR was repeated monthly and gave persistent positive results in detecting SARS-CoV-2 infection. Full genome sequencing was performed on two different patients’ samples collected on 25 February (hCoV-19/Italy/FVG-TS-36474928/2021) and on 24 May 2021 (hCoV-19/Italy/FVG-TS-64028678/2021), and these samples were stored at −80°C until processed, together, in the same analytical session [cycle threshold value (Ct) was S 18, N 19, *Orf1ab* 18 for the first sample and S 17, N 18, *Orf1ab* 17 for the second sample]. In both cases, the B.1.177 variant (Pango v.3.1.20 2022-02-28) was identified (for the materials and method, see **Supplemental**). Interestingly, careful inspection of the sequences identified 21 polymorphisms including the amino-acid T95I (nt 21846) in the spike gene that has been observed in several variants of concern (VOC) including BA.1 (Omicron) (**Table 1**). A reverse mutation was observed in two positions (nt 17333 and 27826). In 13 sites, the frequency of mutated nucleotide ranged from 20% to 59%, suggesting the emergence at different times. In six sites, a polymorphism was detected in both samples, but the proportion of the mutant nucleotide increased in the second sample from 32% to 72%. Excluding the two reverse mutations, the non-synonymous/synonymous mutation ratio was 13/6, suggesting an ongoing positive selection pressure, and this was higher in the *S* gene (7/1) than the *M* gene (2/1) or *ORF1ab* (4/2). The second swab was also inoculated in Vero E6 cells, and viable virus was recovered demonstrating the infectivity of the sample.

**Table 1:** Comparative mutation analysis of the two sequences from patient 1.

Nucleotide position	Ref. seq (nt)	Sample 25/02 (nt)	No. of reads	% mutation	AA	Sample 24/05 (nt)	No. of reads	% mutation	AA	Gene	Mut. type
487	G	G	6,127		Ser	K	7,998	48%	Ser	*ORF1ab*	S
2534	G	G	1,453		Val	R	1,949	50%	Val/Ile	*ORF1ab*	NS
10369	C	C	1,713		Arg	Y	2,018	24%	Arg	*ORF1ab*	S
12561	A	A	4,307		Gln	W	5,300	44%	Gln/Leu	*ORF1ab*	NS
12570	T	T	3,635		Val	K	4,699	57%	Val/Gly	*ORF1ab*	NS
17333	C	Y	4,988	55% (T)	Met/Thr	C	5,691		Thr	*ORF1ab*	NS
21572	T	T	109	4% (C)	Phe	Y	91	76%	Phe/Leu	*S* (S1)	NS
21846	C	C	1,280		Thr	Y	1,216	36%	Thr/Ile	*S* (S1)	NS
21998	C	C	347	9% (T)	His	Y	643	52%	His/Tyr	*S* (S1)	NS
22191	T	T	877		Ile	Y	1,188	49%	Ile/Thr	*S* (S1)	NS
22986	C	C	155		Ala	Y	331	59%	Ala/Val	*S* (RBD)	NS
23009	G	G	155	8%	Val	R	331	40%	Val/Ile	*S* (RBD)	NS
23580	G	G	6,132		Ser	S	5,789	49%	Ser/Thr	*S* (S1)	NS
24034	C	C	234		Asp	Y	289	53%	Asp	*S* (S1)	S
25421	T	T	5,609		Ile	K	5,894	38%	Ile/Ser	*ORF3a*	NS
25728	C	C	759		Val	Y	778	46%	Val	*ORF3a*	S
26527	C	C	146	18% (T)	Ala	Y	269	50%	Ala/Val	*M*	NS
26847	A	A	1,736	2% (T)	Met	W	1,599	52%	Met/Leu	*M*	NS
26939	A	A	1,790		Val	R	1,699	20%	Val	M	S
27826	T	Y	7,485	43% (C)	Met/Thr	T	6,284		Met	*ORF7b*	NS
27972	C	Y	12,915	22% (T)	Gln/Stop	Y	13,408	59% (T)	Gln/Stop	*ORF8*	S

The polymorphisms identified are indicated together with variation percentages. IUPAC codes are used for nucleotides and amino acids. S/NS means synonymous/non-synonymous mutations.

**Figure 1:**
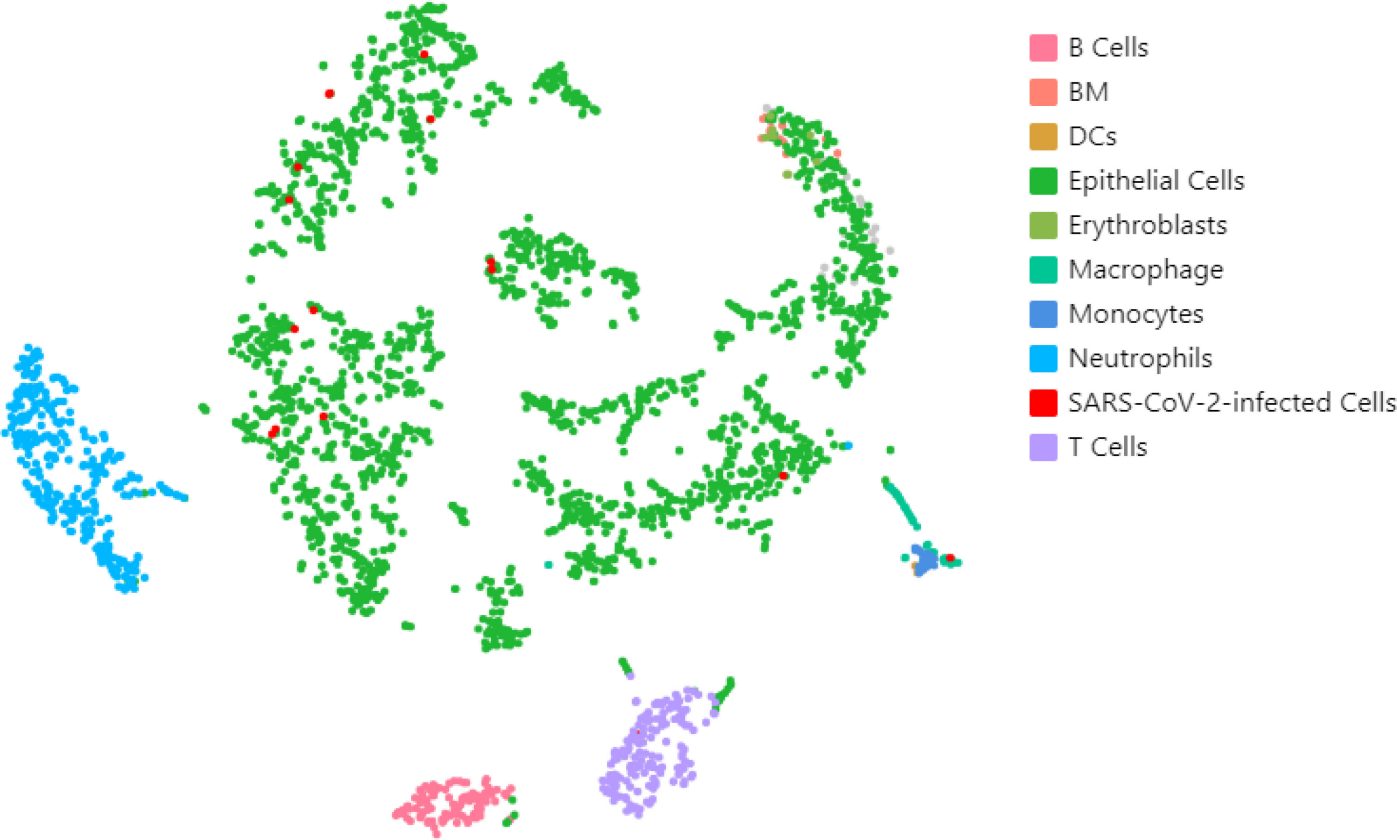
Single-cell transcriptome analysis of nasal brush isolated from a chronic SARS-CoV-2 infection. The two-dimensional t-distributed stochastic neighbor embedding (t-SNE) plot, based on *n* = 2,838 cells, shows the distribution of SARS-CoV-2-infected cells (red dots) into each cluster, representing a different cell type.

Due to the persistence of SARS-CoV-2 infection, in this patient, we investigated the tissue reservoir harboring the infectious virus. Cells obtained by a nasopharyngeal brush in May 2021 were subjected to single-cell RNA sequencing (scRNA-seq). A total of 2,838 cells were recovered from the analysis, which were clustered and classified based on their expression patterns, as shown in **Figure 1**. The most represented cellular types were epithelial cells (*N* = 2,128) and immune cells, including neutrophils (*N* = 304), T cells (*N* = 165), B cells (*N* = 111), macrophages (*N* = 42), and monocytes (*N* = 21). As shown in **Figure 1**, viral RNAs were identified mostly in epithelial cells (red dots, *N* = 14/16), with residual positivity also in macrophages and T cells (*N* = 1 each, respectively).

**Figure 2:**
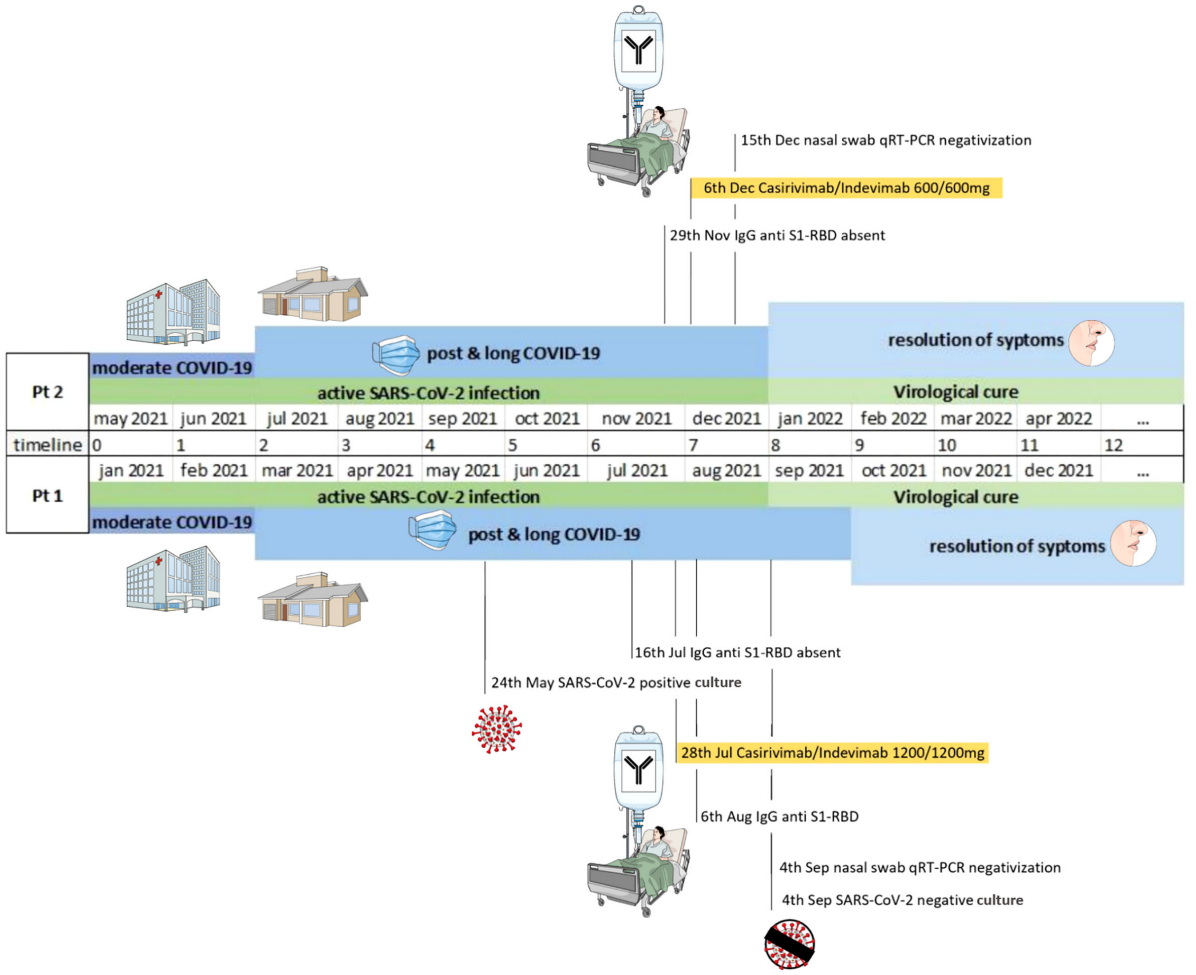
Timeline including the virological course and COVID-19-related symptoms in patients 1 and 2.

The patient did not develop an anti-SARS-CoV-2 humoral response on plasma collected 6 months after the primary infection; more specifically, no IgG anti-S1-RBD was present in July 2021, while only low levels of IgG against the trimeric spike protein were detected. Due to the persistence of upper respiratory tract SARS-CoV-2 infection and symptoms consistent with post-COVID-19 condition, off-labeled anti-SARS-CoV-2 casirivimab/imdevimab (1,200 mg/1,200 mg) was administered intravenously in the outpatient service of the ID unit on July 28. This therapy was well-tolerated without any adverse reaction. After mAb infusion, the patient had a rapid clinical improvement with the resolution of all post-COVID-associated symptoms in the subsequent 3 weeks. On August 6, the patient had positive anti-spike IgG antibodies, confirmed by two independent immunochemiluminescent tests (see **Supplemental**). Anti-trimeric spike protein IgG was positive with 2,080 BAU/ml, as well as IgG anti-S1-RBD with 185,311 AU/ml values. Furthermore, two successive RT-PCR nasal swabs performed on September 4 and 8 did not detect SARS-CoV-2, and the virus could not be isolated in Vero E6 cells. The patient has been doing well during the next 4-month follow-up period. A timeline including the clinical and virological courses is presented in **Figure 2**.

## Case 2

On 17 May 2021, a 67-year-old man presented to the emergency room of our hospital with fever, dyspnea, asthenia, skin, and mucous hemorrhagic manifestations. The patient had a previous diagnosis of CLL (unmutated IGHV gene, positive for 11q deletion with no further alterations of chromosomes 12, 13, and 17 and TP53) that was treated with six cycles of fludarabine, cyclophosphamide, and rituximab (FCR) achieving a complete response in April 2016. Because of progressive symptomatic disease in 2018, he started second-line therapy with ibrutinib with a persistent partial response. During the last 5 years, the patient experienced several bacterial and viral infectious episodes (viral stomatitis, varicelliform herpes zoster with residual neuritis, bacterial infection of the oral cavity, and *Pseudomonas aeruginosa* infection of the nasal cavities). Furthermore, he was vaccinated for COVID-19 with the second dose performed on 9 May 2021 (BNT162b2 mRNA vaccine). At the time of admission to the emergency room, a nasal swab confirmed SARS-CoV-2 infection; blood tests showed moderate anemia (hemoglobin 87 g/L), leukopenia (white blood cells 2.59 × 10 (9)/L, neutrophils 1.08 × 10 (9)/L, lymphocytes 1.45 × 10 (9)/L), severe thrombocytopenia (platelet count 17 × 10 (9)/L), increased acute-phase reactants (C-reactive protein 192.1 mg/L, procalcitonin 2 ng/ml, and D-dimer level 41,791 ng/ml FEU). Oxygen saturation was 92% and a chest X-ray and a pulmonary CT confirmed interstitial pneumonia. The patient was admitted to the ID unit; ibrutinib therapy was stopped, while treatment with amoxicillin and clarithromycin was started together with supplemental oxygen, methylprednisolone therapy, and red blood cell (RBC) and platelet transfusions. During hospitalization, respiratory failure progressively improved and on May 26, he was transferred to a rehabilitation facility with a positive nasal swab for COVID-19. In the following months, he experienced post-COVID-19 condition with exertional dyspnea, asthenia, and hyposthenia of the leg. Due to the progressive increase of lymphocytosis, SARS-CoV-2 infection, and reduced bone marrow reserve, he was persistently pancytopenic requiring RBC and platelet transfusions until July 2021.

A monthly nasal swab for SARS-CoV-2 showed persistent positivity, and on 29 November 2021, serological testing for SARS-CoV-2 was negative (IgG anti-S1-RBD). Full genome sequencing was performed in two different patients’ samples collected on 21 May (hCoV-19/Italy/FVG-GO-36863251/2021) and on 21 July 2021 (hCoV-19/Italy/FVG-GO-36998512/2021), and these samples were stored at –80°C until processed, together, in the same analytical session as described in the methods (see **Supplemental**) (Ct was S 18.2, *Orf1ab* 19.4 for the first sample and N 21, *Orf1ab* 22 for the second sample). The sequences were deposited in the Global Initiative on Sharing All Influenza Data (GISAID) with accession ID EPI_ISL_7015624.2 and EPI_ISL_7015625.2, respectively. In both cases, the variant B.1.1.7 (Pango v.3.1.20 2022-02-28) was identified. Careful analysis of the sequences identified 19 mutations including a 6-nucleotide deletion in positions 22289–22294 (**Table 2**). A reverse mutation was observed in two positions (nt 22337 and 23009), and excluding the reverse mutations and deletion, the non-synonymous/synonymous mutation ratio was 7/3. The second swab was also inoculated in Vero E6 cells, and viable virus was recovered demonstrating the infectivity of the sample.

**Table 2:** Comparative mutation analysis of the two sequences from patient 2.

Nucleotide position	Ref. seq (nt)	Sample 21/05 (nt)	No. of reads	% mutation	AA	Sample 21/07 (nt)	No. of reads	% mutation	AA	Gene	Mut. type
2676	C	Y	538	66%	Pro/Leu	T	7,551	100%	Leu	*ORF1ab*	NS
4230	C	Y	569	70% (T)	Thr/Ile	T	6,051	100%	Ile	*ORF1ab*	NS
5648	A	A	714		Lys	C	8,353	90%	Gln	*ORF1ab*	NS
9515	C	C	693		Leu	T	8,551	100%	Leu	*ORF1ab*	S
9779	T	T	153	15% (A)	Phe/Ile	A	1,476	100%	Ile	*ORF1ab*	NS
13348	G	G	593		Val	T	4,701	89%	Val	*ORF1ab*	S
19862	C	Y	147	31% (T)	Ala/Val	T	1,381	100%	Val	*ORF1ab*	NS
22289	G	G	823		Ala	DEL	12,451	100%	*	*S*	
22290	C	C	821		Ala	DEL	12,452	100%	*	*S*	
22291	T	T	820		Ala	DEL	12,452	100%	*	*S*	
22292	T	T	819		Leu	DEL	12,452	100%	*	*S*	
22293	T	T	819		Leu	DEL	12,458	100%	*	*S*	
22294	A	A	820		Leu	DEL	12,463	100%	*	*S*	
22337	A	W	655	63% (T)	Thr/Ser	A	3,666		Thr	*S*	NS
23009	G	K	123	56% (T)	Val/Lys	G	735		Val	*S*	NS
23012	G	G	123		Glu	A	737	100%	Lys	*S*	NS
25440	G	G	747		Lys	S	10,817	23%	Lys/Asn	*ORF3a*	NS
27720	T	Y	837	47% (C)	Phe	C	10,299	100%	Phe	*ORF7a*	S
27915	G	G	3,830	18% (A)	Gly/Arg	G	54,181		Gly	*ORF8*	NS

The polymorphisms identified are indicated together with variation percentages. IUPAC codes are used for nucleotides and amino acids. S/NS means synonymous/non-synonymous mutations. *ref seq NC_045512.2.

On 6 December 2021, the patient was treated with casirivimab/imdevimab (600mg/600 mg) with good tolerance; a nasal swab for SARS-CoV-2 performed 4 days later was negative. Because of progressive disease, on 30 December 2021, he resumed ibrutinib therapy with no response; on March 2022, BTK and phospholipase Cγ2 (PLCG2) mutations of acquired resistance to ibrutinib therapy were detected. Currently, in April 2022, the patient is in fair clinical conditions, and he started venetoclax therapy. A timeline including the clinical and virological courses is presented in **Figure 2**.

## Discussion

Data from the COVID-19 pandemic have clearly indicated that patients with hematological malignancies are associated with a higher risk to develop multiorgan complications and a significant increased mortality rate upon SARS-CoV-2 infection (3, 4). Because of their immune-incompetent status, secondary to the characteristic of hematological disease and/or treatments adopted to cure it, these patients frequently fail to develop anti-SARS-CoV-2 antibodies or cellular response to primary infection leading to prolonged viral replication (6). The same immune defect leads frequently to an impaired immune response to SARS-CoV-2 vaccination.

Several experiences highlighted this issue; 19 patients with lymphoma treated with chemotherapy regimens, including anti-CD20 antibodies, have been found to show persistent SARS-CoV-2 infection (median duration 65 days, range 3 weeks–12 months), and most of them did not develop anti-SARS-CoV-2 antibodies (16). Similarly, a persistent PCR positivity (defined as SARS-CoV-2 RNA detection ≥30 days after initial positivity) has been observed in 51 (13.9%) of 214 lymphoma patients in a 1-year period of observation. In this series, the risk factors independently associated with prolonged infection were lymphopenia, treatment with anti-CD20 antibodies within 1 year, and cellular therapy including hematopoietic stem cell transplantation (HSCT) (17). Hueso et al. reported a small cohort of lymphoma patients (15) with profound B-cell lymphopenia and prolonged SARS-CoV-2 infection treated with convalescent plasma. The median duration of COVID-19 symptoms was 56 days (range 7–83), and all patients failed to develop SARS-CoV-2 antibodies (18). Other case series described prolonged SARS-CoV-2 infection associated with clinical relapse of COVID-19; for instance, in a patient diagnosed with mantle cell lymphoma (MCL), a blastoid variant was described. He was previously treated with two cycles of bendamustine, cytarabine, and rituximab and experienced persistent SARS-CoV-2 viremia associated with four clinical relapses of COVID-19 effectively treated with remdesivir (19).

On these grounds, the long-term persistent positivity of the SAR-CoV-2 swab observed in our two CLL patients was not surprising. Even though case 1 did not receive any chemotherapy, he had severe hypogammaglobulinemia with the inability to produce neutralizing antibodies (15, 16). As a matter of fact, immunodeficiency in CLL is multifactorial and mediated by T-cell defects, suboptimal complement activity, neutrophil and natural killer cell dysfunction, and altered normal B-cell activity (20). The second patient has been treated with multiple regimens including FCR and, subsequently, ibrutinib due to the progressive disease. Both FCR and ibrutinib are two well-known therapeutic regimens associated with profound immune depression and a higher risk to develop common or opportunistic infections in CLL patients. In particular, patients receiving ibrutinib or, similarly, other BTK inhibitors, which nowadays are more and more adopted as first-line or salvage therapy in CLL, have been associated with a very high risk of COVID-19 complications and mortality (6, 21, 22). The second patient was vaccinated with two doses of mRNA vaccine, the last one on 9 May 2021 before the onset of COVID-19 but unfortunately without achieving active immunization.

Both cases had long complained of symptoms with multiple organ impairment following COVID-19 pneumonia; they did not develop anti-SARS-CoV-2 plasma humoral response and showed persisting positive nasopharyngeal RT-PCR for SARS-CoV-2 for 236 and 199 days, respectively. As suggested by Proal and colleagues (23), SARS-CoV-2 may cause chronic symptoms because it persists in different tissue reservoirs after acute infection as confirmed by the identification of SARS-CoV-2 inert viral RNA and/or proteins. The cell culture obtained by a nasal brush of case 1 detected viral RNA predominantly in epithelial cells but, at a lower extent, also in macrophages and T cells. These findings confirm that SARS-CoV-2 preferentially infects nasal epithelial cells, as already demonstrated by the high levels of angiotensin-converting enzyme 2 (ACE2) expression in this cell type (24). While SARS-CoV-2 infection has been reported also for macrophages by scRNA-seq analysis (25), viral infection of T cells on the contrary has not been reported.

Although data about SARS-CoV-2-acquired mutations in hematological patients are scarce, preliminary experiences demonstrated that persistent viral infection may promote intrahost viral evolution as a consequence of several acquired mutations, particularly in the spike gene. This effect may lead to the emergence of SARS-CoV-2 variants (26, 27) that could negatively impact patients’ clinical outcome, particularly in immunosuppressed hosts. In our two patients, indeed, nasopharyngeal swab samples obtained 2 and 3 months apart showed an increasing number of mutations suggestive of an ongoing positive selection pressure, especially in the *S* gene.

To the best of our knowledge, this is the first analysis reporting the successful outcome of persisting SARS-CoV-2 infection associated with post-COVID-19 condition in hematologic patients treated with anti-SARS-CoV-2 mAbs. Following therapy with casirivimab/imdevimab, nasopharyngeal RT-PCR for SARS-CoV-2 became quickly negative in both patients; at the same time, post-COVID-19 condition symptoms progressively improved until completely disappearing in both patients.

There are two case reports in the literature on SARS-CoV-2 persistent infection treated successfully with mAbs; however, such patients were not found to be affected by long COVID (14, 15). On the contrary, despite the proven effectiveness of monoclonal antibodies, secondary acquired mutations of SARS-CoV-2 following monoclonal antibody therapy are emerging as an immune escape resistance mechanism in patients with B-cell malignancies (28).

At present, there are no evidence-based guidelines indicating how to manage persistent COVID-19 infection in asymptomatic as well as post-COVID-19 patients. Although we cannot exclude that the virological and clinical cure of our patients was just a matter of time, the strong time relationship between mAb infusion and viral disappearance is consistent with the favorable effect of mAb therapy in these patients.

In conclusion, our data show that the recovery of our patients might be due to passive immunity conferred by mAb treatment permitting SARS-CoV-2 clearance and resolution of post-COVID-19 condition. Controlled studies are needed to confirm this therapeutic strategy in immunocompromised patients with persisting viral infection and post-COVID-19 condition. Moreover, the variations observed in the sequences obtained in the two samples collected at 2- and 3-month intervals from both patients suggest a role of persistent SARS-CoV-2 infection as a possible source for the development of viral variants.

## Data availability statement

The datasets presented in this study can be found in online repositories. The names of the repository/repositories and accession number(s) can be found in the article/**Supplementary Material**.

## Ethics statement

This study was reviewed and approved by Regional Ethics Committee (Unique Regional Ethical Committee, Friuli Venezia-Giulia 16 April 2020), No. CEUR 2020-OS-072. Written informed consent for participation was not required for this study in accordance with the national legislation and the institutional requirements.

## Author contributions

LB and OS collected the patient data and wrote the manuscript. PDA analyzed NGS sequencing data and critically revised the manuscript. LS and RK performed SARS-CoV-2 real time PCR and NGS sequencing. PMO performed viral isolation. EO, FD, SDM performed single-cell RNA sequencing. AMa and DL collected and analyzed data and revised the manuscript. FZ and RL critically revised the manuscript and approved the final version of the paper. All the other authors contributed to the article and approved the submitted version.

## Acknowledgments

We thanked Fondazione Cassa di Risparmio di Trieste for the purchase of the Illumina MiSeq sequencer (Chromium 10x Genomics testing) and Generali SpA for the support.

## Conflict of interest

Dr. LB received honoraria for giving lectures at medical meetings from AbbVie. Dr. FZ received advisory board fees or honoraria for giving lectures at medical meetings from Roche, Celgene, Janssen, Sandoz, Gilead, Novartis, AbbVie, Amgen, Sobi, Argenx, Grifols, Takeda, and BeiGene.

The remaining authors declare that the research was conducted in the absence of any commercial or financial relationships that could be construed as a potential conflict of interest.

## Publisher’s note

All claims expressed in this article are solely those of the authors and do not necessarily represent those of their affiliated organizations, or those of the publisher, the editors and the reviewers. Any product that may be evaluated in this article, or claim that may be made by its manufacturer, is not guaranteed or endorsed by the publisher.
