# Supplementary material for: Case report: Long-lasting SARS-CoV-2 infection with post-COVID-19 condition in two patients with chronic lymphocytic leukemia: The emerging therapeutic role of casirivimab/imdevimab
Source: Front Oncol. 2022 Sep 30;12:945060. doi: 10.3389/fonc.2022.945060 (PMC9561900; doi:10.3389/fonc.2022.945060)
Supplement: Supplementary file 1 [file DataSheet_1.docx]

**SUPPLEMENTAL**

**Materials and Methods**

Nasopharyngeal specimens were collected using FLOQSwabs in the universal transport medium (UTM) (COPAN, Brescia, Italy). The first Nasopharyngeal swab at Hospital admittance was tested with Simplexa™ COVID-19 Direct assay (Diasorin, Saluggia, Italy). The following swabs were analyzed either using the Allplex™ 2019-nCoV Assay (Seegene Inc. Seoul, Korea) or with TaqPath™ COVID-19 RT-PCR Kit (Life Technology corporation, Carlsbad, California, USA) following manufacturer instruction. The study was approved by the Regional Ethics Commitee (Unique Regional Ethical Committee, Friuli Venezia-Giulia 16 April 2020), No. CEUR 2020-OS-072.

**Collection and dissociation of cells from a nasopharyngeal swab for scRNA-seq analysis**

Local anesthesia was performed by nebulization of a lidocaine solution into the nostrils of a persistent Covid-19 patient (Case 1) and a brush (CONMED Dispensable Bronchial Cytology Brush) was employed to perform nasal epithelial cells scraping. After each swab, the brush was shaken into a falcon containing 10 ml DMEM/10%FBS to collect nasal cells and rinsed into a second falcon containing 10 ml cleaning solution (PBS). The procedure was repeated three times per nostril. In the Biosafety Level 3 laboratory (BSL-3) at ICGEB, both DMEM/10%FBS and cleaning solution were centrifuged at 400 RCF for 10 minutes. The cell pellets were resuspended with a mix of pre-heated enzymes: 1 ml Corning® Dispase (Sigma-Aldrich, 50 caseynolitic units), 2 ml Acutase (Sigma-Aldrich, 500 units/ml), 500 l DNAse II (Sigma-Aldrich, 2 mg/ml) and incubated for 8 minutes at 37 °C. After incubation, the solutions were gently mixed for at least 20 times and the enzymes inactivated adding 6 ml of Fetal Bovine Serum (FBS) (ThermoFisher). Following cell dissociation, both samples were filtered using a 100 μm cell strainer and pooled. The resulting single-cell suspension was centrifuged at 400 RCF for 10 minutes and the pellet rinsed in DMEM, centrifuged again and resuspended in PBS/0.04% BSA (Roche) for scRNA-seq analysis.

**Single-cell library construction and sequencing**

Samples for single-cell gene expression were processed using a Chromium Single Cell 3′ Library and Gel Bead Kit following the manufacturer’s user guide (10x Genomics). After determination of cell concentration, cells were injected into Chromium Next GEM Chip G. Gel beads-in-emulsion (GEMs) were formed in channels of the chip in the 10x Chromium controller, and then collected in an Eppendorf plate for the GEM-reverse transcription (GEM-RT) reaction. After GEM clean up, GEM-RT products were subjected to cDNA amplification, followed by SPRI select bead clean up. Single-cell libraries were prepared following the manufacturer’s instructions. The resulting cDNA was profiled and quantified using High Sensitivity D1000 ScreenTape Assay for TapeStation Systems (Agilent Technologies). Libraries were sequenced on an Illumina Novaseq 6000 (pair-end sequencing, read length 2x150bp) with a sequencing depth of 1 million reads. Expression analyses and cell clustering classification was performed with Loupe Browser software (10x Genomics).

**Viral genome sequencing**

Total RNA extracted from nasopharyngeal swab, was processed by the Swift Amplicon SARS-CoV-2 research panel for genome amplification and library preparation (Swift Bioscience, USA). High-throughput sequencing was conducted using an Illumina MiSeq sequencer following the standard protocol for paired-end 150-bp reads. The sequencing and data analysis were performed at the Genomic and Epigenomic Laboratory of the AREA Science Park. All tools were run with default parameters unless otherwise specified. The raw sequence data were quality controlled using FastQC v0.11.9 (https://www.bioinformatics.babraham.ac.uk/projects/fastqc/) and Genome assembly was conducted using dedicated Swift dockerized data analysis guidelines as previously reported. (<https://doi.org/10.1128/MRA.01316-20>).

**Serology tests used**

Peripheral venous blood samples were obtained and stored at +4 °C until processing. Serums were tested for SARS-CoV-2 antibodies using the LIAISON SARS-CoV-2 Trimerics IgG chemiluminescent immunoassay (CLIA) (Liason, DiaSorin), a quantitative assay for the detection of IgG antibodies anti-Trimeric Spike glycoprotein of SARS-CoV-2, with a positive cut-off ≥33.8 BAU/mL and an assay range of 4.81–2080 BAU/mL.

Serums were also tested for SARS-CoV-2 antibodies using a quantitative chemiluminescent microparticle immunoassay (CMIA) for the detection of IgG antibodies directed against the receptor-binding domain RBD of the S1 subunit of the SARS-CoV-2 spike protein, with a positive cut-off ≥50.0 AU/mL and an assay range of 6.8–80000 BU/mL.

Two independent tests were run since the likelihood of predicting protective antibody responses is increased when using RBD in the assay, while trimeric IgG are a stronger indicator of virus exposure (GeurtsvanKessel CH Nat Comm 2020, Bonelli F Clin Chem Lab Med 2021)
